# Supplementary material for: Genetic Evidence of Yersinia pestis from the First Pandemic
Source: Genes (Basel). 2025 Jul 31;16(8):926. doi: 10.3390/genes16080926 (PMC12385600; doi:10.3390/genes16080926)
Supplement: Supplementary file 1 [file genes-16-00926-s001.zip › Supplemental Figure S1.pdf]

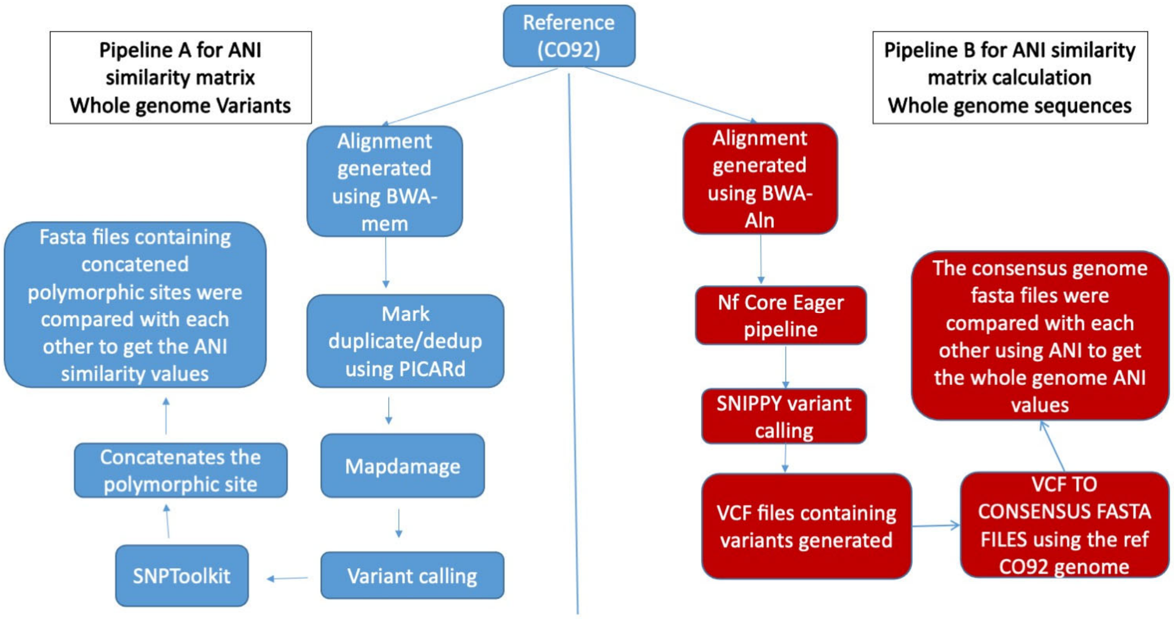

Supplemental Figure S1: Genomic Pipelines for Computing ANI (Average Nucleotide Identity)

The VCF (Variant Call Format) files containing single nucleotide polymorphisms (SNPs) were generated using the snippy-pairwise module of the plague phylogeography pipeline. For the ANI analysis, VCF files for the eight Jerash samples, along with the Tian Shan Hun and VAL001 strains, were utilized. Consensus FASTA sequences were derived from the individual VCF files using the CO92 reference sequence with the consensus utility of bcftools v1.13. Sequence similarity among all samples, including Tian Shan Hun and VAL001 strains, was calculated using Average_nucleotide_identity.py from pyani v0.2.12, based on ANI values.
Pipeline A employed processed variants, including duplicate marking and MapDamage clearance, while Pipeline B used whole genome sequence information for calculations. Both methods produced highly similar results.
